# Dicholine salt of succinic acid, a neuronal insulin sensitizer, ameliorates cognitive deficits in rodent models of normal aging, chronic cerebral hypoperfusion, and beta-amyloid peptide-(25–35)-induced amnesia

**DOI:** 10.1186/1471-2210-8-1

**Published:** 2008-01-23

**Authors:** Zinaida I Storozheva, Andrey T Proshin, Vladimir V Sherstnev, Tatiana P Storozhevykh, Yana E Senilova, Nadezhda A Persiyantseva, Vsevolod G Pinelis, Natalia A Semenova, Elena I Zakharova, Igor A Pomytkin

**Affiliations:** 1 P.K. Anokhin Institute of Normal Physiology, RAMS, Mohovaya 11-4, 125009, Moscow, Russia; 2 Scientific Centre for Children's Health, RAMS, Lomonosovsky prospect 2/62, 119991, Moscow, Russia; 3 Semenov Institute of Chemical Physics, RAS, Kosygina 4, 119991, Moscow, Russia; 4 Institute of General Pathology and Pathophysiology, RAMS, Baltijskaya 8, 125315, Moscow, Russia; 5 Biosignal Ltd., M. Gruzinskaya 29-153, 123557, Moscow, Russia

## Abstract

**Background:**

Accumulated evidence suggests that insulin resistance and impairments in cerebral insulin receptor signaling may contribute to age-related cognitive deficits and Alzheimer's disease. The enhancement of insulin receptor signaling is, therefore, a promising strategy for the treatment of age-related cognitive disorders. The mitochondrial respiratory chain, being involved in insulin-stimulated H_2_O_2 _production, has been identified recently as a potential target for the enhancement of insulin signaling. The aim of the present study is to examine: (1) whether a specific respiratory substrate, dicholine salt of succinic acid (CS), can enhance insulin-stimulated insulin receptor autophosphorylation in neurons, and (2) whether CS can ameliorate cognitive deficits of various origins in animal models.

**Results:**

In a primary culture of cerebellar granule neurons, CS significantly enhanced insulin-stimulated insulin receptor autophosphorylation. In animal models, CS significantly ameliorated cognitive deficits, when administered intraperitoneally for 7 days. In 16-month-old middle-aged C57Bl/6 mice (a model of normal aging), CS enhanced spatial learning in the Morris water maze, spontaneous locomotor activity, passive avoidance performance, and increased brain N-acetylaspartate/creatine levels, as compared to the age-matched control (saline). In rats with chronic cerebral hypoperfusion, CS enhanced spatial learning, passive avoidance performance, and increased brain N-acetylaspartate/creatine levels, as compared to control rats (saline). In rats with beta-amyloid peptide-(25–35)-induced amnesia, CS enhanced passive avoidance performance and increased activity of brain choline acetyltransferase, as compared to control rats (saline). In all used models, CS effects lasted beyond the seven-day treatment period and were found to be significant about two weeks following the treatment.

**Conclusion:**

The results of the present study suggest that dicholine salt of succinic acid, a novel neuronal insulin sensitizer, ameliorates cognitive deficits and neuronal dysfunctions in animal models relevant to age-related cognitive impairments, vascular dementia, and Alzheimer's disease.

## Background

A large body of accumulated evidence suggests that insulin resistance and impairments in cerebral insulin receptor signaling may contribute to age-related cognitive deficits and Alzheimer's disease (AD) [1-10]. The enhancement of brain insulin receptor signaling is, therefore, a promising strategy for the treatment of age-related cognitive disorders. Optimal insulin receptor signaling requires hydrogen peroxide (H_2_O_2_) generated in cells during insulin stimulation [11-17]. The mitochondrial respiratory chain, being involved in insulin-stimulated H_2_O_2 _production, has been identified recently as a potential target for the enhancement of insulin signaling [18]. The rate of insulin-stimulated H_2_O_2 _production depends on the concentration of respiratory substrate, succinate [19].

The aim of the present study is to examine: (1) whether a specific respiratory substrate, dicholine salt of succinic acid (CS), can enhance insulin-stimulated insulin receptor autophosphorylation in neurons, and (2) whether CS can ameliorate cognitive deficits of various origins in animal models.

## Results

### Dicholine salt of succinic acid enhances insulin-stimulated insulin receptor autophosphorylation in neurons

**Figure 1:**
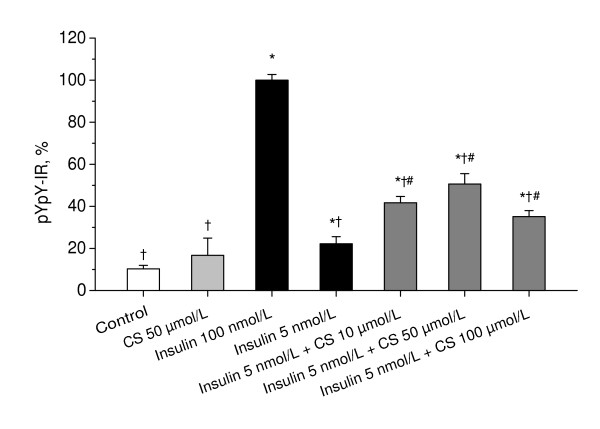
**Effects of CS on the autophosphorylation of insulin receptor in cerebellar granule neurons**. CGN cultures were stimulated with insulin, CS, or combinations of insulin and CS at indicated concentrations for 20 min. Autophosphorylation of insulin receptor was measured as described in Materials and Methods. In each experiment, amount of phosphorylated insulin receptor β-subunit (pYpY-IR) was normalized to total amount of insulin receptor β-subunit and expressed as a percentage of the response produced to 100 nmol/L insulin. Columns represent the means ± SEM of pYpY-IR values obtained from five to nine separate experiments, each performed in duplicate. *P < 0.05 vs. control. ^†^P < 0.05 vs. insulin 100 nmol/L, ^#^P < 0.05 vs. insulin 5 nmol/L.

To examine whether CS enhances the insulin-stimulated autophosphorylation of insulin receptor in neurons, we studied an effect of CS on insulin-stimulated insulin receptor autophosphorylation in a primary culture of rat cerebellar granule neurons (CGN). Figure 1 shows that, whereas by itself, 50 μmol/L of CS does not stimulate insulin receptor autophosphorylation significantly (P = 0.065 vs. control), this concentration of CS significantly enhances the effect of suboptimal concentration of 5 nmol/L insulin (P < 0.001 vs. 5 nmol/L insulin). CS significantly enhances insulin-stimulated insulin receptor autophosphorylation in the range of concentrations from 10 to 100 μmol/L (P < 0.05 vs. 5 nmol/L insulin), although no significant difference is observed between the effects of different concentrations of CS. These results suggest that CS is a neuronal insulin sensitizer, which works in concert with insulin to stimulate insulin receptor autophosphorylation in neurons.

### Dicholine salt of succinic acid improves cognition and neuron functioning in middle-aged mice

**Figure 2:**
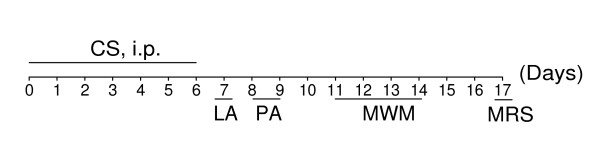
**Experimental schedule of studies in middle-aged C57Bl/6 mice**. Mice were treated with CS (i.p. for 7 days) or saline (i.p. for 7 days, control) and then tested. LA, locomotor activity. PA, step-down passive avoidance. MWM, Morris water maze. MRS, ^1^H-MRS *in vivo*.

To examine whether dicholine salt of succinic acid can ameliorate age-related cognitive deficits, 16-month-old middle-aged C57Bl/6 mice (mean life span of these mice is 26 to 28 months [20]) were treated with CS (1 to 25 mg/kg, i.p.) or saline (control, i.p.) for seven consecutive days. Then, behavioral tests and measurement of whole-brain N-acetylaspartate/creatine (NAA/Cr) ratio by proton magnetic resonance spectroscopy (^1^H-MRS) *in vivo *were carried out on days as indicated in the experimental schedule (Figure 2). 5-Month-old young adult mice were treated with i.p. saline for 7 days.

**Figure 3:**
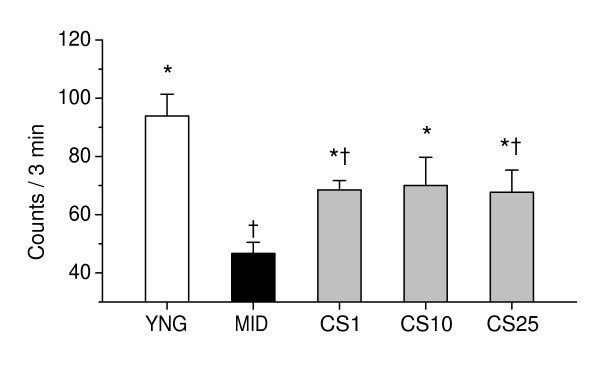
**Effects of CS on spontaneous locomotor activity in middle-aged mice**. Spontaneous locomotor activity in mice was evaluated in the open field test on the day as indicated in the experimental schedule (Figure 2). YNG, young adult mice (i.p. saline for 7 days); MID, middle-aged mice (i.p. saline for 7 days, control); CS1, CS10, and CS25, middle-aged mice treated i.p. for 7 days with CS in doses of 1, 10, or 25 mg/kg respectively. Each group comprised a minimum of eight mice. Columns represent the means ± SEM of locomotor activity counts during a 3-min observation period. *P < 0.05 vs. MID. ^†^P < 0.05 vs. YNG.

As shown in Figure 3, there was a significant difference in spontaneous locomotor activity in the open field between control middle-aged mice and young adult mice (P < 0.001). CS significantly increased the locomotor activity of middle-aged mice, as compared to age-matched controls, when administered in doses of 1–25 mg/kg (P < 0.05).

**Figure 4:**
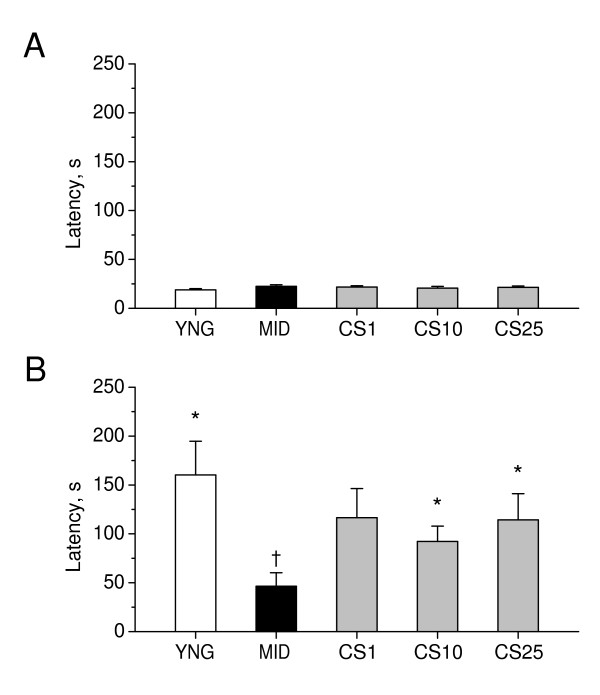
**Effects of CS on learning in the step-down passive avoidance test in middle-aged mice**. Step-down latencies in passive avoidance task were measured during acquisition trial and, 24 hours later, during retention trial on days as indicated in the experimental schedule (Figure 2). A: Acquisition trial. B: Retention trial. YNG, young adult mice (i.p. saline for 7 days); MID, middle-aged mice (i.p. saline for 7 days, control); CS1, CS10, and CS25, middle-aged mice treated i.p. for 7 days with CS in doses of 1, 10, or 25 mg/kg respectively. Each group comprised a minimum of eight mice. Columns represent the step-down latencies means ± SEM. *P < 0.05 vs. MID. ^†^P < 0.05 vs. YNG.

As shown in Figure 4, there was no significant difference in step-down latencies in the passive avoidance test between young adult mice, control middle-aged mice, and CS-treated middle-aged mice on a day of acquisition trial. However, 24 hours later, in the retention test, middle-aged mice exhibited a significant decrease in step-down latencies (P < 0.05) as compared to young adult mice, indicating a learning deficit induced by aging. CS increased retention latencies in middle-aged mice, as compared to age-matched controls, when administered in doses of 10 and 25 mg/kg (P < 0.05). These data suggest that CS significantly improves passive avoidance learning in middle-aged mice, as compared to the age-matched controls.

**Figure 5:**
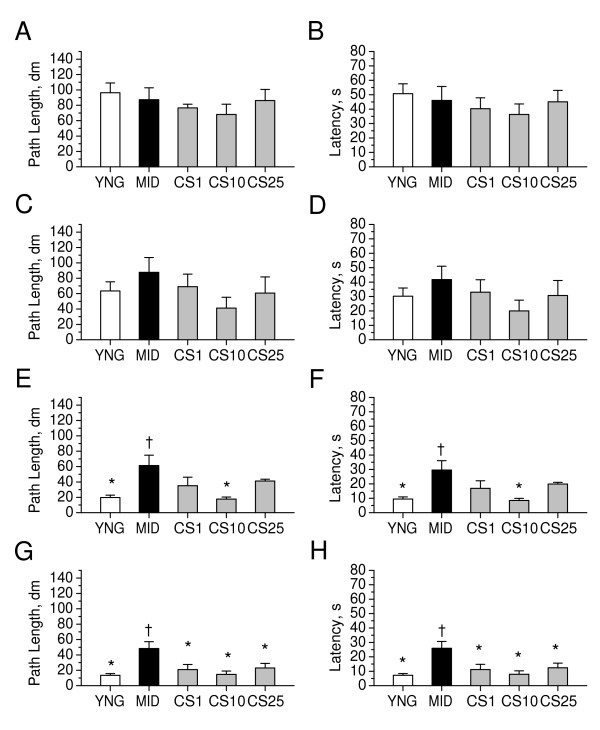
**Effects of CS on spatial learning in the water-maze test in middle-aged mice**. Path lengths and latencies to escape to the hidden platform in water maze were measured during the 4-day training period on days as indicated in the experimental schedule (Figure 2). A, B: The day 1 of the trial. C, D: The day 2 of the trial. E, F: The day 3 of the trial. G, H: The day 4 of the trial. YNG, young adult mice (i.p. saline for 7 days); MID, middle-aged mice (i.p. saline for 7 days, control); CS1, CS10, and CS25, middle-aged mice treated i.p. for 7 days with CS in doses of 1, 10, or 25 mg/kg respectively. Each group comprised a minimum of eight mice. Columns represent the path lengths or latencies means ± SEM. *P < 0.05 vs. MID. ^†^P < 0.05 vs. YNG.

As shown in Figure 5, both path length and latency to escape to the hidden platform in the Morris water maze decreased progressively (i.e., learning was progressive) during the 4-day training period in all groups of mice. Two-way ANOVA revealed significant day effect in all experimental groups (P < 0.01). There was a significant difference, however, between middle-aged mice and young adult mice on the third day (P < 0.01 for both measures) and the fourth day (P < 0.001 for both measures) of the training, indicating that spatial learning in middle-aged mice was slower, as compared to young adult mice. Two-way ANOVA revealed significant group effect across days (F1,16 = 26.9, P < 0.01) and group × day interaction (F1,3,70 = 7.51, P < 0.05), when groups of young adult and middle-aged mice were compared. CS significantly decreased the escape latencies in middle-aged mice, as compared to age-matched controls, at the fourth day of the training, when administered in doses of 1–25 mg/kg (P < 0.05). Two-way ANOVA revealed significant effect for CS doses of 1 mg/kg (F1,14 = 8.47, P < 0.05) and 25 mg/kg (F1,15 = 7.26, P < 0.05) across days and significant both group effect (F1,14 = 10.48, P < 0.05) and group × day interaction (F1,1,62 = 6.79, P < 0.05) for dose of 10 mg/kg. CS significantly decreased the path lengths in middle-aged mice, as compared to age-matched controls, on the day 4 of training, when administered in doses of 1–25 mg/kg (P < 0.05). Two-way ANOVA revealed significant group effects for CS doses of 1 mg/kg (F1,14 = 8.01, P < 0.05) and 25 (F1,15 = 6.95 P < 0.05) across days and significant both group effect (F1,14 = 9.84, P < 0.05) and group × day interaction (F1,1,62 = 7.01, P < 0.05) for CS dose of 10 mg/kg. These data suggest that CS significantly improved spatial learning in middle-aged mice, as compared to the age-matched controls.

**Figure 6:**
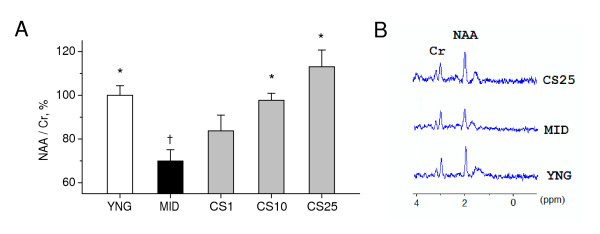
**Effects of CS on brain NAA/Cr levels in middle-aged mice**. NAA/Cr levels in brains of mice were measured by ^1^H-MRS *in vivo *on day as indicated in the experimental schedule (Figure 2). A: Brain NAA/Cr levels. YNG, young adult mice (i.p. saline for 7 days); MID, middle-aged mice (i.p. saline for 7 days, control); CS1, CS10, and CS25, middle-aged mice treated i.p. for 7 days with CS in doses of 1, 10, or 25 mg/kg respectively. Each group comprised a minimum of eight mice. Columns represent the NAA/Cr means ± SEM expressed as a percentage of the mean NAA/Cr in brains of young adult mice. *P < 0.05 vs. MID. ^†^P < 0.05 vs. YNG. B: Representative ^1^H MRS spectra from control young adult mice (YNG), middle-aged mice (MID), and middle-aged mice treated with CS in dose of 25 mg/kg (CS25). Signal assignment: NAA, methyl protons of NAA; Cr, methyl protons of Cr.

N-Acetylaspartate/creatine (NAA/Cr) ratio is widely believed to be a reliable noninvasive marker of neuronal function and viability in the adult brain. As shown in Figure 6, middle-aged mice exhibited a significant 30% drop in whole-brain brain NAA/Cr levels (P < 0.001) by data of ^1^H-MRS *in vivo *study, as compared to young adult mice, thus indicating age-related decline in neuronal function. When administered in doses of 10 and 25 mg/kg, CS significantly increased whole-brain NAA/Cr levels in middle-aged mice, as compared to the age-matched controls (P < 0.01). These data suggest that CS significantly improved neuron functioning in middle-aged mice.

### Dicholine salt of succinic acid improves cognition and neuron functioning in rats with chronic cerebral hypoperfusion

**Figure 7:**
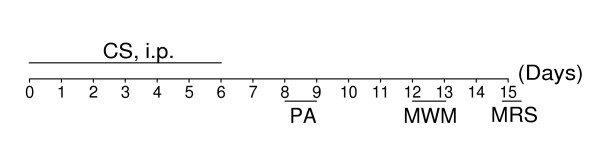
**Experimental schedule of studies in 2VO rats**. Rats were undergone to bilateral occlusion of the common carotid arteries (2VO) and three hours later the treatment was started. Rats received CS or saline (control) i.p. for 7 days and then tested. PA, step-through passive avoidance. MWM, Morris water maze. MRS, ^1^H-MRS *in vivo*.

To examine whether CS can ameliorate cognitive deficits induced by chronic cerebral hypoperfusion, rats with permanent bilateral carotid artery occlusion (two-vessel occlusion, 2VO) were treated with CS (1 to 25 mg/kg, i.p.) or saline (control) for seven consecutive days, and a battery of tests were carried out on days as summarized in the experimental schedule (Figure 7). Sham-operated rats were treated with i.p. saline for 7 days.

**Figure 8:**
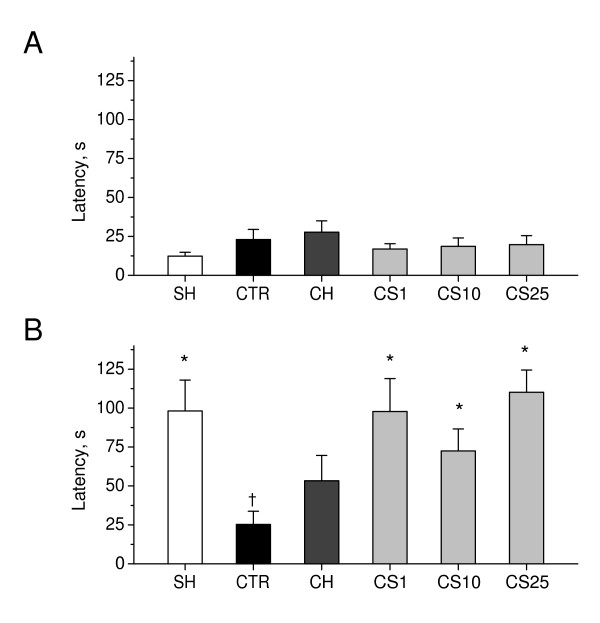
**Effects of CS on learning in the step-through passive avoidance test in 2VO rats**. Step-through latencies in passive avoidance task were measured during acquisition trial and, 24 hours later, during retention trial on days as indicated in the experimental schedule (Figure 7). A: Acquisition trial. B: Retention trial. SH, sham-operated rats (i.p. saline for 7 days); CTR, 2VO rats (i.p. saline for 7 days, control); CH, 2VO rats treated i.p. for 7 days with choline chloride in dose of 10 mg/kg; CS1, CS10, and CS25, 2VO rats treated i.p. for 7 days with CS in doses of 1, 10, or 25 mg/kg respectively. Each group comprised a minimum of nine rats. Columns represent the step-through latencies means ± SEM. *P < 0.05 vs. CTR. ^†^P < 0.05 vs. SH.

As shown in Figure 8, there was no significant difference in step-through latencies to enter the dark compartment in passive avoidance test between sham-operated rats, control 2VO rats, and CS-treated 2VO rats on the day of acquisition trial. However, 24 hours later, in the retention test, 2VO rats exhibited significant decrease in step-through latencies as compared to sham-operated rats (P < 0.01), indicating a learning deficit induced by chronic cerebral hypoperfusion. CS significantly increased retention latencies in 2VO rats, as compared to control 2VO rats, when administered in doses of 1–25 mg/kg (P < 0.05). These data suggest that CS significantly improved passive avoidance learning in rats with chronic cerebral hypoperfusion.

**Figure 9:**
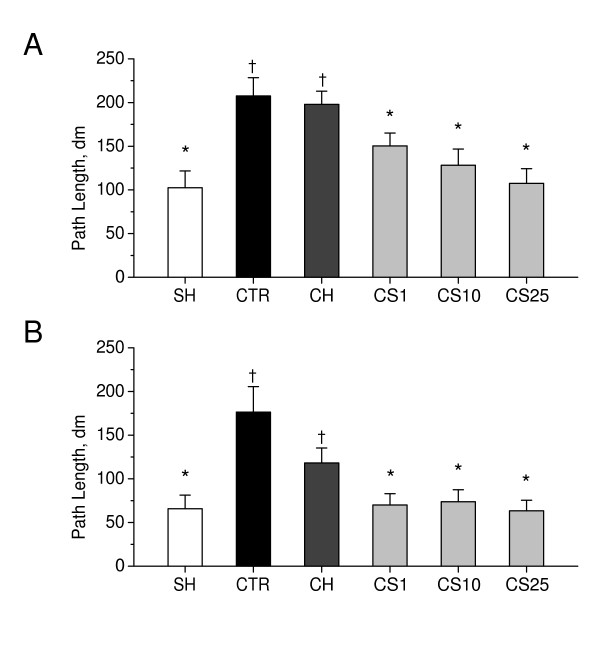
**Effects of CS on spatial learning in the water-maze test in 2VO rats**. Path lengths to escape to the hidden platform in water maze were measured during the 2-day training period on days as indicated in the experimental schedule (Figure 7). A: The day 1 of the trial. B: The day 2 of the trial. SH, sham-operated rats (i.p. saline for 7 days); CTR, 2VO rats (i.p. saline for 7 days, control); CH, 2VO rats treated i.p. for 7 days with choline chloride in dose of 10 mg/kg; CS1, CS10, and CS25, 2VO rats treated i.p. for 7 days with CS in doses of 1, 10, or 25 mg/kg respectively. Each group comprised a minimum of eight rats. Columns represent the path lengths means ± SEM. *P < 0.05 vs. CTR. ^†^P < 0.05 vs. SH.

Figure 9 shows that path length to escape to the hidden platform in Morris water maze task decreased during the 2-day training period, in all groups of rats. There was, however, a significant difference between control 2VO rats and sham-operated rats on the first day and the second day of training (P < 0.01), indicating impairments in spatial learning induced by chronic cerebral hypoperfusion. CS significantly decreased the path lengths in 2VO rats, as compared to control 2VO rats, on day 1 and, particularly, on day 2 of training, when administered in doses of 1–25 mg/kg (P < 0.01). These data suggest that CS significantly improved spatial learning in rats with chronic cerebral hypoperfusion.

**Figure 10:**
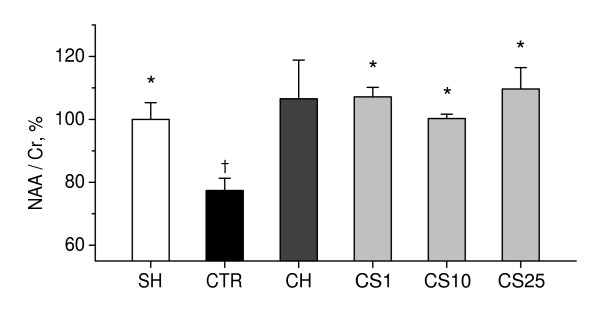
**Effects of CS on brain NAA/Cr ratios in 2VO rats**. NAA/Cr levels in brains of rats were measured by ^1^H-MRS *in vivo *on day as indicated in the experimental schedule (Figure 7). SH, sham-operated rats (i.p. saline for 7 days); CTR, 2VO rats (i.p. saline for 7 days, control); CH, 2VO rats treated i.p. for 7 days with choline chloride in dose of 10 mg/kg; CS1, CS10, and CS25, 2VO rats treated i.p. for 7 days with CS in doses of 1, 10, or 25 mg/kg respectively. Each group comprised from five to nine animals. Columns represent the NAA/Cr means ± SEM expressed as a percentage of the mean NAA/Cr in brains of sham-operated rats. *P < 0.05 vs. CTR. ^†^P < 0.05 vs. SH.

As shown in Figure 10, 2VO rats exhibited a significant 22% decrease in whole-brain NAA/Cr levels, as compared to sham-operated rats (P < 0.001), indicating impairments in neuronal function induced by chronic cerebral hypoperfusion. CS significantly increased whole-brain NAA/Cr levels in 2VO rats, as compared to controls, when administered in doses of 1–25 mg/kg (P < 0.05).

There was no significant difference between 2VO rats treated with choline chloride (10 mg/kg, i.p. for 7 days) and control 2VO rats (saline, i.p. for 7 days) in passive avoidance performance (P = 0.10) and in spatial learning (P = 0.11). Hence, choline chloride, a reference choline compound, showed no significant therapeutic effect on cognitive deficits in 2VO rats. However, MRS data indicate a role of choline in normalization of NAA/Cr deficits.

### Dicholine salt of succinic acid improves learning and increases activity of brain choline acetyltransferase in rats with β-amyloid peptide-(25–35)-induced amnesia

**Figure 11:**
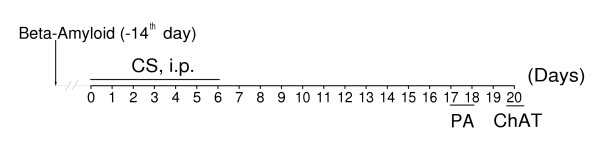
**Experimental schedule of studies in β-amyloid peptide-(25–35)-induced rats**. Rats were undergone to a single injection of β-amyloid peptide-(25–35) into brain NBM. Two weeks later, rats were treated with CS or saline (control) i.p. for 7 days and then tested. PA, step-through passive avoidance. ChAT, choline acetyltransferase measurement.

To examine whether CS can ameliorate cognitive deficits induced in rats by a single injection of β-amyloid peptide-(25–35) into the brain *nucleus basalis magnocellularis *(NBM), the β-amyloid peptide-(25–35)-induced rats were treated with CS (1 to 25 mg/kg, i.p.) or saline (control) for seven consecutive days. Sham-operated rats induced by a single injection of saline into the brain NBM were treated with i.p. saline for 7 days. Step-through passive avoidance test and measurement of activity of choline acetyltransferase (ChAT) in brain cortex homogenates were carried out on days, as indicated in the experimental schedule (Figure 11).

**Figure 12:**
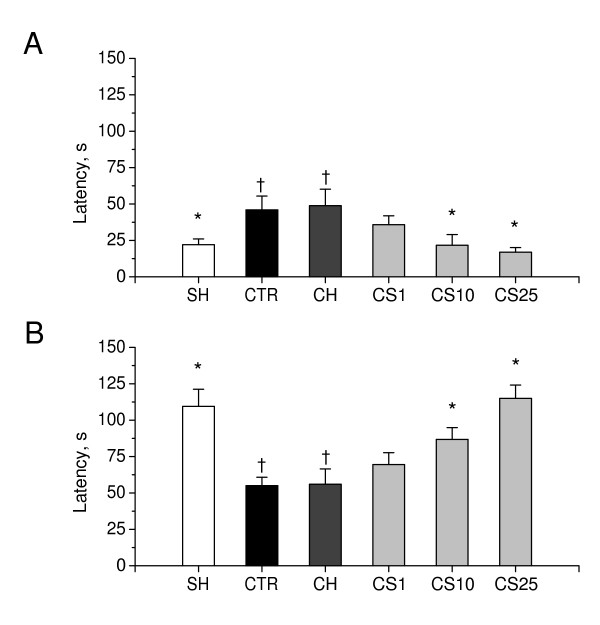
**Effects of CS on learning in the step-through passive avoidance test in rats with β-amyloid peptide-(25–35)-induced amnesia**. Step-through latencies in passive avoidance task were measured during acquisition trial and, 24 hours later, during retention trial on days as indicated in the experimental schedule (Figure 11). A: Acquisition trial. B: Retention trial. SH, sham-operated rats (i.p. saline for 7 days); CTR, β-amyloid peptide-(25–35)-induced rats (i.p. saline for 7 days, control); CH, β-amyloid peptide-(25–35)-induced rats treated i.p. for 7 days with choline chloride in dose of 10 mg/kg; CS1, CS10, and CS25, β-amyloid peptide-(25–35)-induced rats treated i.p. for 7 days with CS in doses of 1, 10, or 25 mg/kg respectively. Each group comprised eight rats. Columns represent the step-through latencies means ± SEM. *P < 0.05 vs. CTR. ^†^P < 0.05 vs. SH.

As shown in Figure 12, there was a significant difference in step-through latencies to enter the dark compartment in passive avoidance test between sham-operated rats and control β-amyloid peptide-(25–35)-induced rats, on the day of acquisition (P < 0.05), and 24 hours later, on the day of retention trials (P < 0.01). On the day of acquisition trials, CS significantly decreased step-through latencies, as compared to the controls, when administered in doses of 10 and 25 mg/kg (P < 0.01). On the day of retention trials, CS significantly increased step-through latencies, as compared to the control, when administered in doses of 10 and 25 mg/kg (P < 0.01). These data suggest that CS significantly improves passive avoidance learning in rats with β-amyloid peptide-(25–35)-induced amnesia.

As shown in Table 1, there was a significant 27% decrease in ChAT activity in the control β-amyloid peptide-(25–35)-induced rats, as compared to sham-operated rats (P < 0.001), indicating cholinergic dysfunction induced by β-amyloid peptide-(25–35) toxicity. CS significantly increased ChAT activity in rats with β-amyloid peptide-(25–35)-induced amnesia, when administered in the highest studied dose of 25 mg/kg (P < 0.05 vs. control).

**Table 1:** Effect of CS on brain ChAT activity in rats with β-amyloid peptide-(25–35)-induced amnesia.

Treatment Groups	ChAT activity, dpm/mg of tissue
SH	6.15 ± 0.21*
CTR	4.52 ± 0.25^†^
CH	4.84 ± 0.68
CS1	5.19 ± 0.35
CS10	5.39 ± 0.41
CS25	5.72 ± 0.38*

ChAT activity in brain cortex homogenates was assayed by radiometry on the day as indicated in the experimental schedule (Figure 11). SH, sham-operated rats (i.p. saline for 7 days); CTR, β-amyloid peptide-(25–35)-induced rats (i.p. saline for 7 days, control); CH, β-amyloid peptide-(25–35)-induced rats treated i.p. for 7 days with choline chloride in dose of 10 mg/kg; CS1, CS10, and CS25, β-amyloid peptide-(25–35)-induced rats treated i.p. for 7 days with CS in doses of 1, 10, or 25 mg/kg respectively. Each group comprised eight rats. Data represent the ChAT activity means ± SEM expressed as dpm/mg of wet tissue. *P < 0.05 vs. CTR. ^†^P < 0.05 vs. SH.

Choline chloride (10 mg/kg, i.p.), the reference choline compound, showed no significant effect on passive avoidance learning and cerebral ChAT activity in rats with β-amyloid peptide-(25–35)-induced amnesia, when administered for 7 days.

## Discussion

In the present study we identified a highly effective treatment of cognitive deficits of various origins with dicholine salt of succinic acid, the neuronal insulin sensitizer.

Initially, we examined whether CS can work as an insulin sensitizer in the brain. It is generally accepted that insulin signaling requires autophosphorylation of the insulin receptor kinase at tyrosine residues in the activation loop of the kinase domain [21-27]. Upon autophosphorylation, the receptor undergoes a major conformational change, resulting in unrestricted access of protein substrates and ATP to the kinase active site and an approximate two-order increase in the kinase activity [28-30]. In the present study, we demonstrate that, although CS alone does not stimulate insulin receptor autophosphorylation significantly, it significantly enhances the response to the suboptimal insulin concentration. The effect of the combination of insulin and CS is much greater than sum of effects of insulin and CS taken alone. These results suggest that CS is a neuronal insulin sensitizer, which works in concert with insulin to stimulate insulin receptor autophosphorylation in neurons.

Dicholine salt of succinic acid is a chemical substance, but its components, choline and succinate, are naturally occurring metabolites widely distributed in mammalian tissues. Average concentrations of succinate and choline in the blood of healthy humans at rest are 1–9 μmol/L [31] and 7–10 μmol/L [32] respectively. During physical exercises or severe hypoxia, levels of succinate and choline increase markedly [33,34]. For example, breath-hold dives for 1 min and treadmill running increases venous succinate in humans to 125 and 93 μmol/L respectively [33]. This means that CS is an effective insulin sensitizer at physiologically occurring concentrations, typically observed in mammals during hypoxia or apneic work.

We further investigated the effects of intraperitoneal administration of CS on cognition and neuronal function in animals with cognitive deficits of various origins.

To examine whether CS, the neuronal insulin sensitizer, can ameliorate cognitive deficits induced by normal aging, experiments with middle-aged C57Bl/6 mice were carried out. These mice represent a model of a mild cognitive deficit associated with age-related changes in the expression of genes, including genes with possible roles in synaptic plasticity and learning [35], while neuronal loss with aging is relatively rare in mice of this strain [20]. We demonstrate that CS significantly improves passive avoidance learning, spontaneous locomotor activity, and spatial learning in middle-aged mice, as compared to age-matched controls. To test whether CS can ameliorate neuronal dysfunction in middle-aged mice, ^1^H-MRS *in vivo *study was carried out in addition to tests listed above. It is generally accepted that the brain level of N-acetylaspartate (NAA), frequently expressed as N-acetylaspartate/Creatine ratio, is a reliable noninvasive marker of neuronal integrity and functioning, since NAA is localized almost exclusively to neurons in the adult brain and NAA decrease is closely correlated with neuronal loss or neuronal dysfunction [36-41]. Reversible NAA deficits reflect a reversible state of neuronal dysfunction preceding neuronal loss [42-45]. NAA quantification is, therefore, considered to be a valuable tool for assessing the effects of potential neuroprotective therapies. In this study, age-related NAA deficits in middle-aged mice were reversed after the treatment with CS, indicating the neuroprotective effect of CS. Taken together, results in middle-aged mice demonstrate that CS ameliorates both age-related cognitive deficits and neuronal dysfunction caused by normal aging. These CS effects were long-lasting beyond the seven-day treatment period and were found to be significant about two weeks following the treatment.

To determine whether CS, the neuronal insulin sensitizer, can ameliorate cognitive deficits induced by chronic cerebral hypoperfusion, experiments with 2VO rats were carried out. Although originally described as a rat model for vascular dementia, the chronic cerebral hypoperfusion induced by permanent bilateral carotid artery occlusion is relevant to Alzheimer's disease, since there is also a slowly developing reduction in cerebral blood flow in this condition [46]. The two-vessel occlusion causes a chronic decrease in the cerebral blood flow to 52–64% of the original level [47] and induces progressive and long-lasting cognitive deficit, cholinergic dysfunction, and progressive neuronal damage in the brain [48,49]. In this study, we found that CS significantly improved passive avoidance learning and spatial learning in 2VO rats, as compared to the control 2VO rats. NAA deficits in brains of 2VO rats were also reversible on the treatment with CS. Thus, CS ameliorates both cognitive deficits and neuronal dysfunction in 2VO rats. These CS effects were long-lasting, extending beyond the seven-day treatment period, and were found to be significant ten days following the CS treatment.

There is evidence that β-amyloid, a hallmark of Alzheimer's disease, can interfere with insulin receptor signaling [50], whereas insulin stimulates clearance and degradation of β-amyloid and thus prevents β-amyloid accumulation in the brain [51-53]. Activity of choline acetyltransferase, a key enzyme of acetylcholine biosynthesis, is reduced in insulin receptor-positive neurons in Alzheimer's disease and expression of choline acetyltransferase is increased with insulin stimulation [54]. These findings provide a rationale for the use of insulin and insulin sensitizers in the treatment of cognitive deficits caused by β-amyloid toxicity. In the present study, we examined the effect of CS, the neuronal insulin sensitizer, on cognitive deficits induced in rats by a single injection of β-amyloid peptide-(25–35) into brain *nucleus basalis magnocellulari*s. Such injection causes behavioral dysfunctions, impairs learning and memory, and disrupts cortical cholinergic innervations, thus modeling Alzheimer's disease [55]. Also, β-amyloid peptide-(25–35) decreases activity of brain choline acetyltransferase, a key enzyme of acetylcholine biosynthesis [56]. In the present study, we demonstrate that CS significantly improves passive avoidance learning and increases ChAT activity in the brains of β-amyloid peptide-(25–35)-induced rats. The CS effects lasted beyond the seven-day treatment period and were found to be significant two weeks following the treatment.

Choline chloride, the reference choline compound, was used in the present study to discriminate the effects of choline and succinate moieties in the CS molecule. As compared to highly effective CS treatment, choline chloride demonstrated no significant effects on cognitive performance in 2VO rats and rats with β-amyloid peptide-(25–35)-induced amnesia. However, MRS data reveal a role for choline in normalization of NAA/Cr deficits in brain of 2VO rats. This indicates that although CS effects relates mainly to the action of succinate, the neuroprotective effect of CS is caused, at least in part, by the presence of choline moiety in the CS molecule.

Earlier, several lines of evidence have suggested that treating insulin resistance might improve cognitive function. It has been reported that rosiglitazone, a peripheral insulin sensitizer and potent PPAR full agonist, attenuates learning and memory deficits in Tg2576 Alzheimer mice [57]. In a small pilot study, rosiglitazone appeared to significantly improve the cognitive ability of AD patients [58]. In a Phase II clinical study, rosiglitazone was found to improve the cognitive ability of mild to moderate AD patients [59]. It has been proposed that PPARγ agonism induces neuronal mitochondrial biogenesis and improves glucose utilization leading to improved cellular function and provides mechanistic support for the improvement in cognition observed in treatment of Alzheimer's patients with rosiglitazone [60].

In general, the results of the present study support the idea that targeting insulin receptor signaling in neurons may help to reduce both cognitive deficits and neuronal dysfunctions associated with aging and Alzheimer's disease.

## Conclusion

The results of the present study suggest that dicholine salt of succinic acid, the novel neuronal insulin sensitizer, ameliorates cognitive deficits and neuronal dysfunctions in animal models relevant to age-related cognitive impairments, vascular dementia, and Alzheimer's disease.

## Methods

### Materials

Dicholine succinate salt (2:1), formula [(CH_3_)_2_NCH_2_CH_2_OH]_2_^+^·^-^OOCCH_2_CH_2_COO^-^, was prepared by a reaction of succinic acid with choline base in the Russian Scientific Center on Drug Safety (Staraya Kupavna, Moscow region). PhosphoDetect™ Insulin Receptor (pTyr1162/1163) ELISA kit and Insulin Receptor (β-Subunit) ELISA Kit were from Calbiochem. Other materials were purchased from Sigma, ICN, Gibco, Biosource, Molecular Probes, or Acros.

### Animals

Male Wistar rats and C57Bl/6 mice were from the Laboratory of Biological Trials of the Pushchino Branch of Shemyakin-Ovchinnikov Institute of Bioorganic Chemistry (Pushchino, Moscow Region). Animals were housed in groups of 4 per cage at a constant temperature 21°C in a light-controlled room at 14/10 light-dark cycle. Food and water were freely available. All animal studies were carried out in accordance with the requirements of our institutional committees for the keeping and use of laboratory animals and in accordance with the "Principles of Laboratory Animal Care" formulated by the National Institutes of Health. All animals were allocated to experimental groups randomly, using computer-generated random numbers.

### Neuronal culture

Cerebellar granule neurons (CGN) were prepared from 7- to 8-day-old Wistar rats as described [61,62]. Cerebellum was dissected and placed in ice-cold Ca^2+^/Mg^2+^-free Hanks' buffered saline (HBSS) without Phenol Red (Gibco). After mincing the tissue with fine scissors, the tissue was placed in Ca^2+^/Mg^2+^-free HBSS with Phenol Red and 0.1% trypsin for 15 min at 36°C. Trypsin was inactivated by washing with normal HBSS. Cells were dissociated by trituration and pelleted in HBSS. Then, the cells were resuspended in Neurobasal Medium (Gibco) supplemented with B-27 Supplement (Gibco), 20 mmol/L KCl, GlutaMax (Gibco) and penicillin/streptomycin and plated with density 5 × 10^6 ^cells/ml onto 35 mm × 10 mm sterile cell culture dishes which had been previously coated with poly-D-lysine. The cultures were maintained at 36°C in a humidified atmosphere of 5% CO_2 _and 95% air and fed with supplemented Neurobasal Medium. Cultures were treated on day 3 with 10 μmol/L cytosine arabinoside (Sigma) for 24 h to prevent glial proliferation. CGN at 7 to 8 days were used for experiments.

### Insulin receptor phosphorylation assay

Amounts of double phosphorylated β-subunit of insulin receptor (pYpY-IR) were measured by PhosphoDetect™ insulin receptor (pTyr1162/1163) ELISA kit (Calbiochem) suitable for studies with rat insulin receptor. CGN cultures were incubated in Hepes-buffered salt solution (145 mmol/L NaCl, 5.6 mmol/L KCl, 1.8 mmol/L CaCl_2_, 1 mmol/L MgCl_2_, 20 mmol/L HEPES, and 5 mmol/L glucose) at pH 7.4 for 30 min, followed by exposure to vehicle, insulin, CS, or combinations of insulin with CS for 20 min. The experiment was terminated by removing the medium, washing with ice-cold PBS, and adding 120 μL per dish cell lysis buffer (Biosource) supplemented with 1 mmol/L PMSF, 50 mmol/L protease inhibitor set III (Sigma), and 2 mmol/L sodium ortovanadate as the inhibitor of tyrosine phosphatases at 4°C for 20 min. Lysates were centrifuged at 12,000 rpm at 4°C for 12 min. In each CGN lysate, pYpY-IR amounts were measured as described by the manufacturer's manual. Obtained values were normalized to total amounts of insulin receptor β-subunit (IR) measured by insulin receptor (β-subunit) ELISA kit (Calbiochem). The results are expressed as a percentage of the response produced to 100 nmol/L insulin.

### Two-vessel occlusion

Experimental cerebral hypoperfusion was induced in rats by permanent bilateral occlusion of the common carotid arteries (two-vessel occlusion, 2VO) as described [47]. The rats were anesthetized with sodium pentobarbital (40 mg/kg, i.p.) and the common carotid arteries of the rat were separated from the cervical sympathetic and vagal nerves through a ventral cervical incision. Then, the arteries were ligated with silk sutures. The same surgical procedure was performed in the sham-operated rats but without the actual ligation.

### β-Amyloid peptide-(25–35) injection

β-Amyloid peptide-(25–35)-induced amnesia in rats was induced as described previously [55]. The rats were anesthetized with sodium pentobarbital (30 mg/kg, i.p.) and β-amyloid peptide-(25–35) was injected bilaterally into *nucleus basalis magnocellularis *of rat brain as a sterile solution of 2 μg per 1 μL of saline per side through the guide cannula with Hamilton microsyringe according to stereotaxic coordinates: AP -1.5, DL ± 2.7, and H 8.1 [63]. Sham-operated rats were injected bilaterally into NBM with 1 μL of saline.

### Behavioral tests

Spontaneous locomotor activity of mice was evaluated in open field tests in an automated mode using the Opto-Varimex-3 (Columbus Instruments, OH) photocell-based activity monitor. The Opto-Varimex-3 animal activity monitor employs a photocell beam grid. Animals were placed individually into the activity monitor and spontaneous locomotor activity (total accumulated counts of a horizontal single photocell interruption) was collected for a 3-min period.

The apparatus for step-down passive avoidance test consisted of a box (22 × 24 × 27 cm) with a stainless-steel grid floor. A circular Plexiglas platform (diameter, 8 cm; height, 2 cm) was fixed at the center of the box. During the training, each mouse was placed individually on the platform. When the mouse stepped down from the platform and placed its four paws on the grid floor, an electric shock 1.0 mA was delivered for 3 s. The retention trial was carried out twenty-four hours after the training session in a manner similar to the training except that no electric shock was delivered *via *grid floor. Each mouse was placed again on the platform, and step-down latency was recorded until 180 s had elapsed.

A step-through box for passive avoidance test consisted of a light compartment connected to a dark compartment by a controllable door. This test consisted of two trials. In the acquisition trial, the rats were individually placed into the light compartment, the door to a dark compartment was opened, and the latency until the rat entered the dark compartment was recorded. After the rat had stepped through the door, the door was closed and an electric shock 0.8 mA was delivered for 1 s *via *the grid floor. After receiving the footshock, the rat was returned to a home cage. The retention trial was carried out twenty-four hours after the acquisition trial. In the retention trial, each animal was placed into the light compartment, and the step-through latency was recorded until 180 s had elapsed.

The water maze test was performed as described by Morris [64]. The experimental apparatus consisted of circular water pool (diameter, 120 cm; height, 60 cm) containing water at 24°C to a depth of 40 cm and rendered opaque by adding milk. A Plexiglas escape platform (8 × 8 cm for mice or 10 × 10 cm for rats) was submerged 1.5 cm (for mice) or 2 cm (for rats) below the water surface and placed at the midpoint of one quadrant. The location of the platform remained the same throughout the training period. The pool was located in a test room containing various prominent visual cues. Six training trials per day were conducted with an inter-trial interval of 2 min. Animals were placed in the pool at one of six starting positions. In each training trial, the time and path length required to escape onto the hidden platform was recorded. Results of six training trials were averaged to obtain a single representative value, and the averages were used for final statistics. Animals that found the platform were allowed to remain on the platform for 30 s and were then returned to the home cage during the inter-trial interval. Animals that did not find the platform within 120 s were softly guided to the platform for 30 s at the end of the trial.

All behavioral experiments were carried out by investigators blinded to treatment groups.

### ^1^H-MRS measurements *in vivo*

Spectra were recorded at ^1^H frequency 400 MHz using the Bruker AM-400 WB spectrometer (Bruker, Reinsretten, Germany) with a vertical magnet equipped with a home-build probe of outer diameter 70 mm. An animal under pentobarbital sodium anesthesia (40 mg/kg, i.p.) was fixed in the probe head, the surface coil (6 mm in diameter for mice or 14 mm in diameter for rats) being positioned directly onto the skull at animal's sinciput. Magnetic field homogeneity was optimized by the water signal. Line widths of 40 to 90 Hz were routinely obtained. The ^1^H-MRS spectra were accumulated and processed as described [65,66]. Metabolite ratios of NAA/Cr were calculated from the relative peak height measurements using the spectrometer's software. All ^1^H-MRS measurements were carried out by investigator blinded to treatment groups.

### Choline acetyltransferase assay

Rats were decapitated under sodium pentobarbital (40 mg/kg, i.p.) anesthesia. Brains were quickly removed and homogenized. ChAT activity in cerebral cortex homogenates was measured by the method described by Fonnum [67]. All ChAT measurements were carried out by investigator blinded to treatment groups.

### Statistics

Data were analyzed for statistical significance by one-way analysis of variance (ANOVA). Data of the water maze test were analyzed for statistical significance by two-way ANOVA. Values are given as means ± SEM. Differences were considered significant at P < 0.05.

## Abbreviations

AD, Alzheimer's disease; ANOVA, analysis of variance; ChAT, choline acetyltransferase; CGN, cerebellar granule neurons; Cr, creatine; CH, choline chloride; CS, dicholine salt of succinic acid; HBSS, Hanks' buffered salt solution; HEPES, 4-(2-hydroxyethyl)-1-piperazineethanesulfonic acid; ^1^H-MRS, proton magnetic resonance spectroscopy; i.p., intraperitoneal; NAA, N-acetylaspartate; NBM, *nucleus basalis magnocellularis*; PBS, phosphate-buffered saline; PMSF, phenylmethylsulfonyl fluoride; SEM, standard error of mean; 2VO, two-vessel occlusion.

## Competing interests

The author(s) declare that they have no competing interests.

## Authors' contributions

ZIS carried out surgical operations, behavioral tests, and the data analysis in animal studies. ATP carried out surgical operations and behavioral tests in animal studies. VVS participated in the design of behavioral studies, critical intellectual discussion, and manuscript evaluation/critique. TPS carried out the *in vitro *studies with CGN cultures and data analysis. YES carried out the *in vitro *studies with CGN cultures and data analysis. NAP carried out the *in vitro *studies with CGN cultures and data analysis. VGP participated in the design of the *in vitro *studies with CGN cultures, critical intellectual discussion, and manuscript evaluation/critique. NAS carried out the ^1^H NMR study *in vivo *and the data analysis. EIZ carried out the measurement of ChAT activity and the data analysis. IAP conceived, designed and coordinated the study, and drafted the manuscript. All authors read and approved the final manuscript.
